# Supplementary material for: Providers perspectives on a team-based maternal health care delivery in Ghana: A qualitative study
Source: PLOS Glob Public Health. 2025 Jun 4;5(6):e0004246. doi: 10.1371/journal.pgph.0004246 (PMC12136346; doi:10.1371/journal.pgph.0004246)
Supplement: Attachment — Submitted filename: IPC Addressing Comments.docx [file pgph.0004246.s002.docx]

Dear Editor,

Kindly, find below a point-by-point response to the comments shared by the reviewers and yourself, to help bring our manuscript to an appreciable standard.

| **Comment** | **Response** | **Page** |
| --- | --- | --- |
| **Reviewer 1 (Corrections highlighted in yellow colour)** | | |
| 1. Some literature on disrespectful experiences of mothers seeking maternity care in Ghana in the introduction would be helpful in justifying the need for this study.  2. Any new knowledge this study adds to literature should clearly be captured in the conclusion.   3. An omission identified in line 26 '' Of health care providers in a collaborative team-based setting at a health ''FACILITY'' in Ghana.  4. omission in line 473 ''maternal care in Ghana '' IN'' ensuring .... | Additional literature reviewed on ‘disrespectful experiences of mothers seeking maternity care in Ghana’ as part of the introduction as advised.  New knowledge captured in conclusion as advised.  Introduction restructured for improvement, with all omissions addressed.  Introduction restructured for improvement, with all omissions addressed. | 4, 5  24  2-7 |
| **Reviewer 2 (Corrections highlighted in yellow colour)** | | |
| Overall, this paper is excellent, timely and crucial in light of the increased advocacy happening around human rights in childbirth. It is well written and adopts a unique approach, i.e. interprofessional team-based approach to improving the quality of care.  The abstract: Authors may want to strengthen the abstract by structuring it in a way that clearly shows that the paper looks at how interprofessional team-based care is supposed to contribute to respectful and dignified care. When reading the abstract, the connection between these two themes is separated as more emphasis is laid on interprofessional team-based care alone and not on how interprofessional team-based care is supposed to lead to respectful and dignified care.  Lines 25 and 26 feel a bit incomplete. 51 and 52 need restructuring to avoid repetition. 54, meanwhile, may not be the most appropriate transition phrase for that sentence  You make use of both these terms: Interdisciplinary vs interprofessional- Not sure whether they are meant to refer to the same thing. Consistency is important  In the conclusion and recommendation section, the authors may need to be a bit more clear with regard to who is being called upon to address the structural barriers. While it is not wrong to ask the facilities to address structural issues, the problem runs deeper than that. Most structural issues affecting service provision fall within the state’s duty to promote, protect and fulfil the right to health. Placing this responsibility on facilities may be a bit misleading. The same applies to issues of hospital staffing and continuous professional development. Who exactly is responsible for this between the state and the facility? see Sadler, M., Santos, M. J., Ruiz-Berdún, D., Rojas, G. L., Skoko, E., Gillen, P., & Clausen, J. A. (2016). Moving beyond disrespect and abuse: addressing the structural dimensions of obstetric violence. Reproductive health matters, 24(47), 47–55. [https://doi.org/10.1016/j.rhm.2016.04.002](https://doi.org/10.1016/j.rhm.2016.04.002);  [https://www.ohchr.org/sites/default/files/Documents/Publications/Factsheet31.pdf](https://www.ohchr.org/sites/default/files/Documents/Publications/Factsheet31.pdf)  While the authors adopt the language of respectful and dignified care, they may want to familiarise themselves with the language of Respectful Maternity Care. This may be a strategic approach to strengthen this body of work further as it provides an avenue to adopt a rights-based framing for certain issues raised in the paper. For example, the authors raise concerns about privacy and confidentiality, which are human rights issues that have been discussed in the Respectful Maternity Care Charter by the White Ribbon Alliance. To be truly holistic, an approach to improving women's experience during pregnancy, childbirth, and the postpartum period must Incorporate a rights-based lens. The spirit of RMC, which authors call respectful and dignified care, is about centring the birthing person’s human rights in providing care. | Thank you.  Abstract has been re-organized to reflect the nexus between interprofessional team-based settings and the provision of respectful and dignified maternal care.  Introduction has been improved and re-organized with all repetitions addressed, to appropriately reflect the study topic.  Introduction has been improved and re-organized, with the confusion between ‘interdisciplinary vs interprofessional’ appropriately addressed. Although interdisciplinary and interprofessional’ had different connotations, we decided to replace ‘interdisciplinary’ with ‘multidisciplinary’ to forestall any confusion.  Conclusion and implications section re-organized, to clearly spell out the novel knowledge contributed by the study as well as holistic recommendations for primary stakeholders responsible for dealing with the systemic barriers to respectful and dignified maternal care identified, guided by Sadler et al. (2016) as suggested.  The human rights prescriptions of the Respectful Maternity Care (RMC) Charter acknowledged and referenced in the re-organized background, discussion, conclusion and recommendation, as suggested. | 1, 2  6  24, 25  5, 22, 25 |
